# Propagation of Disturbances in AC Electricity Grids

**DOI:** 10.1038/s41598-018-24685-5

**Published:** 2018-04-24

**Authors:** Samyak Tamrakar, Michael Conrath, Stefan Kettemann

**Affiliations:** 1 Department of Physics and Earth Sciences, Jacobs University, Campus Ring 1, 28759 Bremen, Germany; 2 Institute of Physics, Carl von Ossietzky Universität Oldenburg, Ammerländer Heerstraße 114-118, Oldenburg, 26129 Germany; 3 Division of Advanced Materials Science, Pohang University of Science and Technology (POSTECH), San 31, Hyoja-dong, Nam-gu, Pohang, 790-784 South Korea

## Abstract

The energy transition towards high shares of renewable energy will affect the stability of electricity grids in many ways. Here, we aim to study its impact on propagation of disturbances by solving nonlinear swing equations describing coupled rotating masses of synchronous generators and motors on different grid topologies. We consider a tree, a square grid and as a real grid topology, the german transmission grid. We identify ranges of parameters with different transient dynamics: the disturbance decays exponentially in time, superimposed by oscillations with the fast decay rate of a single node, or with a smaller decay rate without oscillations. Most remarkably, as the grid inertia is lowered, nodes may become correlated, slowing down the propagation from ballistic to diffusive motion, decaying with a power law in time. Applying linear response theory we show that tree grids have a spectral gap leading to exponential relaxation as protected by topology and independent on grid size. Meshed grids are found to have a spectral gap which decreases with increasing grid size, leading to slow power law relaxation and collective diffusive propagation of disturbances. We conclude by discussing consequences if no measures are undertaken to preserve the grid inertia in the energy transition.

## Introduction

In order to cover the increasing human energy demand by renewable energy resources and to ensure that this energy will be available wherever and whenever it is needed, more efficient energy transport and storage technologies need to be developed. The fluctuations in generated power by wind turbines and solar cells - both in time and geographically - demand to explore new strategies to store energy on all time scales and to distribute the power in the grid smartly. At the same time, the spreading of critical disturbances throughout the grid has to be prevented to ensure the stability of the entire grid. Renewable energy resources fluctuate strongly in time on time scales as small as seconds. Moreover the inverter-connected wind turbines and solar cells provide no inertia^1^. This is in contrast to conventional generators, whose rotating masses hold inertia and thereby momentary power reserve available for the grid, which makes the grid resilient and prevents strong fluctuations of the grid frequency on time scales of several seconds^2,3^. As the inertia in the grid keeps decreasing with higher share of renewables, the grid is responding on shorter time scales to disturbances. It is therefore essential to understand the impact of this development on the stability of electricity grids. In this article, we aim to find out if and how the relaxation and propagation of disturbances in AC grids is modified when the grid inertia from the rotating masses of generators is decreasing. We focus on the spreading of weak disturbances, which do not cause by themselves a failure of transmission lines or generators.

The dynamic interaction and response of generators and consumers is studied modeling the grid as a network of nonlinear oscillators^2–7^. These nonlinear dynamic power balance equations, so called swing equations, describe the dynamics of coupled rotating masses by a system of coupled differential equations for local rotor angles *ϕ*_*i*_, where *i* denote the grid nodes. We aim to analyze the propagation of disturbances in AC grids on short time scales up to several seconds. Therefore, we do not consider control measures, which typically set in on longer time scales^3^. We expect that a better understanding of this short time transient dynamics will then contribute to the optimization of primary and secondary control measures^1–3^. Furthermore, we expect that the understanding of the spreading of small disturbances will be important for the design of improved power system stabilizers (PSS). Thereby it can contribute to prevent the occurrence of larger disturbances, whose spreading could result in overload and failure of transmission lines, and cause cascading failures. We note that the dynamics of cascading failures involves additional mechanisms, such as the overheating of transmission lines. While most studies of cascading failures are based on stationary power balance calculations or simplified topological flow models such as the messenger model^8–14^ and our own work based on nonlinear power flow calculations^15^, only few model their dynamics^16,17^, introducing for example a time scale for overheating of transmission lines.

In this article, we solve the nonlinear swing equations numerically and explore how a local perturbation propagates throughout the grid. The origin of disturbances can be fluctuations in generating power or sudden changes of transmission line capacitance. We analyze these results, employing analytical results obtained from a linear response theory, mapping the swing equations on discrete linear wave equations for small perturbations^18^. Depending on the geographical distribution of power, power transmission capacity and grid topology we find that the disturbance may either decay exponentially in time with the decay rate of a single oscillator Γ^0^, or exponentially with a smaller decay rate Γ < Γ^0^, or, even more slowly, decaying with a power law in time. Such a slow power law decay is found to arise together with slow, diffusive propagation^18^.

## Model Description

### Grid Topologies

**Figure 1:**
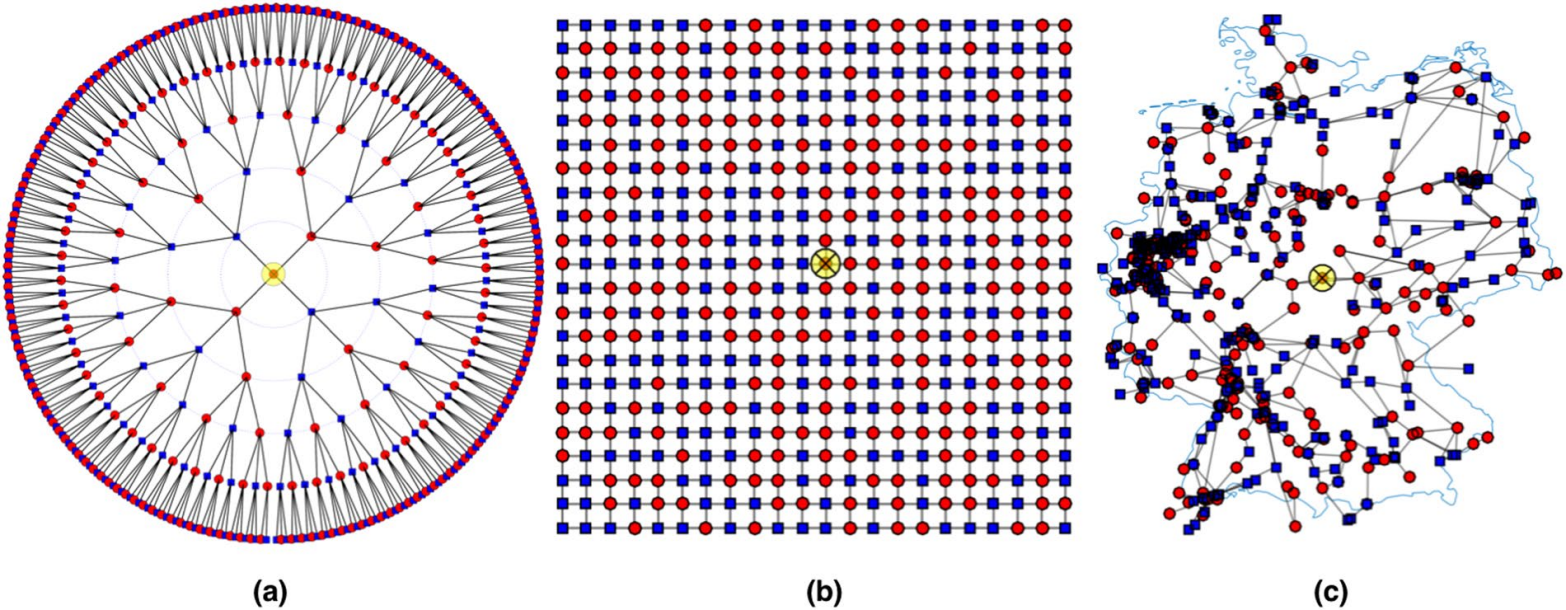
Grid topologies: (**a**) Cayley tree, *l* = 5 branching levels, branching number *b* = 3, *N* = 484 nodes and 483 links. (**b**) *L* = 22 square grid with *N* = 484 nodes, 924 links, random arrangement of generators and consumers. (**c**) German transmission grid with *N* = 502 nodes, 673 links^19^. The size of the square and tree grid is chosen to be comparable to the German grid. Red circles represent generators, blue squares motors. The yellow circle marks the node where a perturbation is applied.

**Figure 2:**
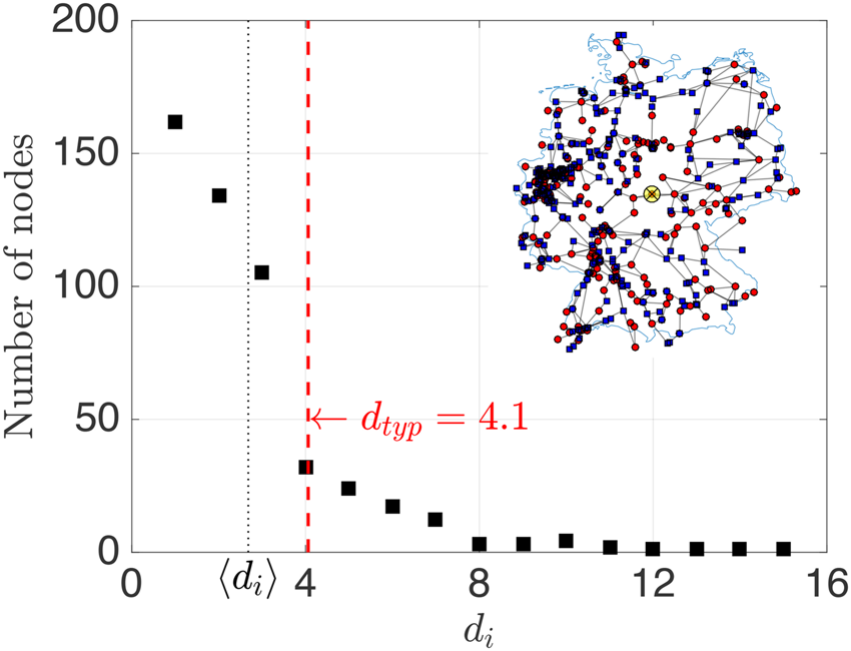
Distribution of node degree *d*_*i*_ for German transmission grid.

Aiming at a systematic approach, we consider three different grid topologies. The Cayley tree, Fig. 1(a), resembles distribution grids which are operated in a tree-like fashion to pinpoint and repair failures more easily. Branches grow outward from a central node with branching number *b*, forming *l* branching levels. Tree grids are characterized by the distance between neighbored nodes *a*, the total number of nodes *N*, branching number *b* and level *l*. The degree *d*_*i*_, the number of links connecting node *i* to any other node is an important network characteristic. For Cayley trees it is *d* = *b* + 1 except at outer edge nodes where *d* = 1. The square grid, Fig. 1(b), is a meshed grid, used as basic model for transmission grids with their strict redundancy demand to guarantee continuing operation when a single line fails (n-1 criterion), characterized by distance *a*, linear grid size *L* and number of nodes *N* = (*L*/*a*)^2^. Their degree is *d* = 4, except at edges (*d* = 3) and corners (*d* = 2). Thirdly, we use the open-source SciGRID dataset^19^ of the German transmission grid, Fig. 1(c), as a real-world example of a highly meshed grid. Excluding island nodes, the largest connected network of the three highest voltage levels, 400 kV, 380 kV, 220 kV and some 110 kV lines has *N* = 502 nodes and 673 links. Its degree *d*_*i*_ has a wide distribution, Fig. 2, with average degree 〈*d*_*i*_〉 = 2.7 and typical degree ${d}_{typ}=\sqrt{\langle {{d}_{i}}^{2}\rangle }=\mathrm{4.1.}$ Excluding singly connected nodes (which are mostly artifacts since the data set does not include the transnational European grid) we get an average degree 〈*d*_*i*_〉 = 3.5.

### Dynamic AC Grid Model

AC transmission grids are three-phase systems which are typically loaded and operated symmetrically, so that the power flow balance equations depend on a single phase, only. Neglecting Ohmic losses along the lines, which are small in high voltage transmission grids, we assume purely inductive transmission lines with combined inductance *L*_*ij*_ between nodes *i* and *j* with power capacity *K*_*ij*_ = |*V*_*i*_||*V*_*j*_|/(*ωL*_*ij*_) where *V*_*i*_, *V*_*j*_ are the voltages at nodes *i* and *j*. *ω* = 2*πf* with grid frequency *f* = 50 Hz. We assume constant voltage amplitude *V* throughout the grid, *V*_*i*_ = *V* exp(ı*Φ*_*i*_). Since voltage amplitudes change typically on larger time scales than phases, we will focus on the dynamics of phases *Φ*_*i*_. Fixing the voltage *V* there are no dynamic terms in the reactive power balance equation (they appear in higher order when voltage dynamics in addition to the phase dynamics is considered^3^), so that we need to consider active power balance equations, only. Since our main goal is to study the influence of grid topology and inertia on the phase dynamics we assume equal inductance *L* and power capacity *K* = *V*^2^/(*ωL*), for all links, yielding the power capacity between nodes *i* and *j* as *K*_*ij*_ = *KA*_*ij*_, where *A*_*ij*_ is the grid adjacency matrix. Thereby, the stationary active power flow balance equations are obtained from Kirchhoff’s laws as^20^

1 $${P}_{i}=K\sum _{j}{A}_{ij}\,\sin ({{\varphi }}_{i}-{{\varphi }}_{j}),\,\,\sum {P}_{i}=0.$$

However, as loads and generated power vary in time, that power balance may become violated and the nodal phases become dynamic. Denoting the solution of the stationary balance equation, equation (1) by the phase shift ${\theta }_{i}^{0},$ we can write

2 $${{\varphi }}_{i}(t)=\omega t+{\theta }_{i}^{0}+{\alpha }_{i}(t),$$

where *α*_*i*_(*t*) are the dynamic phase shifts. The grid nodes are either connected to synchronous generators with inertia *J*_*i*_ or to loads which can be motors with a finite inertia, being modeled as synchronous motors^3,21^. The electric power *P*_*i*_, is either positive (generator) or negative (motor). In order to be able to study the dependence on system parameters and topology we take homogenous parameters *P*_*i*_ = *s*_*i*_*P*, with *s*_*i*_ ∈ {+, −} and *J*_*i*_ = *J*. The phase dynamics is governed by the balance of changes in kinetic energy, energy dissipation and electric power exchange with adjacent grid nodes, yielding the swing equations^4–6,21^.

3 $${P}_{i}=(\frac{J}{2}\frac{{\rm{d}}}{{\rm{d}}t}+\gamma ){(\frac{{\rm{d}}{{\varphi }}_{i}}{{\rm{d}}t})}^{2}+\sum _{j}{F}_{ij},$$

where *γ* is a damping constant, *F*_*ij*_ = *K*_*ij*_ sin(*Φ*_*i*_ − *Φ*_*j*_) the power flow in the transmission line between nodes *i* and *j*.

If the nodes would not be coupled by the transmission lines, the phase at each node would decay exponentially fast with the local relaxation time *τ* = *J*/*γ*, which increases with inertia *J* and decreases with damping parameter *γ*. Therefore, when studying the temporal and spatial dependence of *α*_*i*_, it is convenient to scale the time with relaxation time *τ*. Inserting equation (2) into equation (3) one finds for small phase velocities, ${\partial }_{t}{\alpha }_{i}\ll \omega ,$ the swing equations,

4 $${\tau }^{2}{\partial }_{t}^{2}{\alpha }_{i}+2\tau {\partial }_{t}{\alpha }_{i}={s}_{i}{{\rm{\Pi }}}_{P}-{{\rm{\Pi }}}_{K}\,\sum _{j}{A}_{ij}\,\sin ({\theta }_{i}^{0}-{\theta }_{j}^{0}+{\alpha }_{i}-{\alpha }_{j}),$$

with dimensionless parameters Π_*P*_ = *JP*/(*γ*^2^*ω*) and Π_*K*_ = *JK*/(*γ*^2^*ω*).

## Results

### Transient Dynamics

In order to study the transient behavior of AC grids perturbed by local disturbances, we solve the nonlinear swing equation (4) on the different grid topologies of Fig. 1 as function of the set of parameters (*τ*,Π_*K*_, Π_*P*_).

#### Stationary solution

Before any perturbation is applied, we calculate the stationary state ${\theta }_{i}^{0}$ at every node *i* in the grid. This is accomplished by first obtaining a solution of equation (1) for small phase differences linearizing $\sin ({\theta }_{i}^{0}-{\theta }_{j}^{0})\to {\theta }_{i}^{0}-{\theta }_{j}^{0}\mathrm{.}$ Thereby equation (1) can be rewritten by introducing the weighted graph Laplacian matrix *H*_*ij*_ = −*K*_*ij*_ + *δ*_*ij*_∑_*l*_ *K*_*il*_, as

5 $${P}_{i}=\sum _{j}{K}_{ij}({\theta }_{i}^{0}-{\theta }_{j}^{0})\Rightarrow P=H\cdot {\theta }^{0},$$

where **P** and *θ*^0^ are vectors, whose *i* -th component is the power and stationary phase at node *i*, respectively. *H* has at least one Eigenvalue zero. Therefore, we need the pseudo inverse *H*^+^, yielding *θ*^0^ = *H*^+^ ⋅ **P**. We use this solution as initial condition for a numerical root solver to find the solution of the nonlinear equation, equation (1). This way, the numerical accuracy of the stationary solution is maximized to make sure that we use as initial condition for the swing equations the stationary state.

#### Numerical Solution of Swing Equations

**Figure 3:**
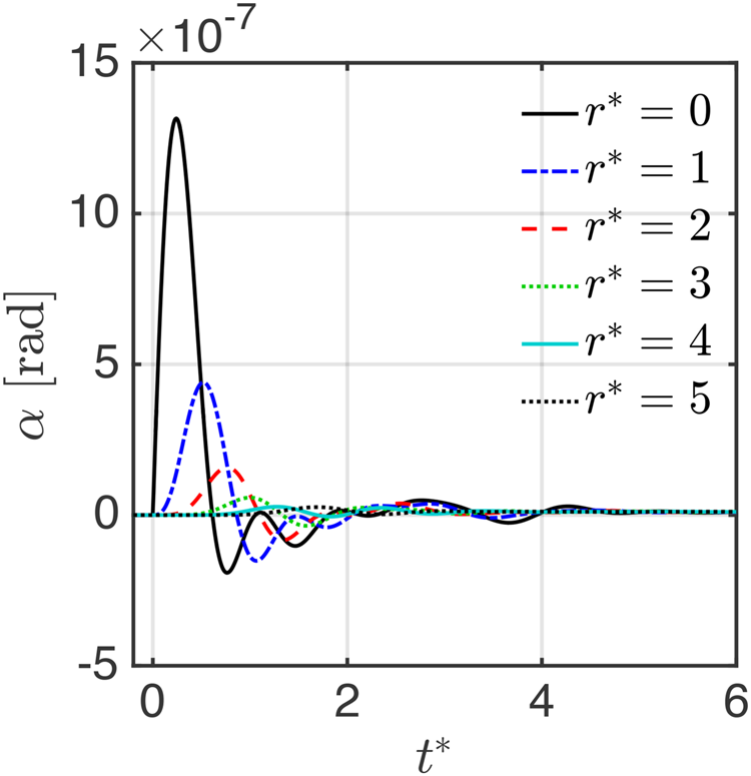
Phase perturbation *α*(r^*^, t^*^ = *t*/*τ*) for *b* = 3 Caylee tree grid at distances *r* = r^*^/a (Π_*K*_ = 10, *σ* = Π_*P*_/Π_*K*_ = 0.08).

Having found the stationary phases ${\theta }_{i}^{0}$ as solutions of equation (1), we insert them into swing equation (4). Next, we solve these equations when the AC grid is perturbed by a local disturbance as outlined in detail in Supplementary I. As disturbance of the stationary state we increase the power for a short time interval 0 ≤ *t* ≤ Δ*t*_*pert*_ at the grid node marked with ‘x’ Fig. 1. We choose as small perturbing power one per mille of the initial generator power *P*. The resulting transient behavior of phase deviations *α*(*t*) is shown in Fig. 3 for the Cayley tree grid of Fig. 1(a) as function of rescaled time t^*^ = *t*/*τ* for parameters (Π_*K*_ = 10, *σ* = Π_*P*_/Π_*K*_ = 0.08). We define the distance between any two nodes ${r}_{ij}^{\ast }$ as the number of lines in the shortest path connecting them^22^. We see that the phase at the disturbed node, r^*^ = 0 is perturbed first, reaching a maximum after a delay time, decaying then in an oscillatory manner. Phases of nodes further away from the origin of the disturbance are perturbed later and reach smaller amplitudes. We analyze this temporal and spatial propagation of the perturbation quantitatively below. Since we are interested in the propagation of small disturbances which do not destabilize the system, we review next the conditions for stability in order to make sure that we choose the size of the disturbance accordingly.

### Stability

Without disturbance, the criterion for stable (allowed) and unstable (forbidden) parameters Π_*P*_, Π_*K*_ is the existence of a non-complex solution to stationary state equation (1). Thus, the ratio *σ* = Π_*P*_/Π_*K*_ determines whether parameters are allowed. A critical value *σ*_*c*_ exists which may depend on grid topology and power distribution. If there are no clusters of consumers or clusters of generators, the critical value at node *i* is given by *σ*_*ci*_ = *d*_*i*_, where *d*_*i*_ is the node degree. Thus, the critical value, below which the whole grid is stable, is given by *σ*_*c*_ = min(*d*_*i*_). For a general distribution of *P*_*i*_ there can be clusters of generators or consumers and the critical value *σ*_*c*_ is determined by the size and form of these clusters. If a cluster of generators has total power *P*_*C*_ = ∑_cluster_ *P*_*i*_, with effective degree *d*_*C*_, as obtained by counting the number of consumers which are directly coupled to that cluster, the critical value is given by *σ*_*c*_ = min(*P d*_*C*_/*P*_*C*_). As outlined in the article in Supplementary section II the damping term and therefore the parameter *τ* = *J*/*γ* determines the size of the basin of attraction, if a stable fixed point exists, that is for *σ* < *σ*_*c*_. Outside of the basin of attraction the system is attracted to unstable fixed points, so called limit cycles. For large damping there can be a regime where there is no coexistence with limit cycles and the whole phase space is stable, see f.e. refs^23,24^ for a review. Therefore, depending on the magnitude *α* of the perturbation it can destabilize the grid already at σ^*^(*α*) < *σ*_*c*_. In the Supplementary section II. we derive a typical upper limit for the size of the perturbation *α* before it kicks the system out of the stable region and find that the disturbance indeed destabilizes the grid already at values σ^*^(*α*) < *σ*_*c*_. Only in the limit, when the perturbation amplitude is vanishing we recover σ^*^(*α* → 0) = *σ*_*c*_.

### Classification of Transient Dynamics: Parametric Phase Diagrams

In order to analyze the transients we calculate absolute values of the power flow change in the transmission line between nodes *i* and *j*, ${\rm{\Delta }}{F}_{ij}(r)=|{F}_{ij}(r)-{F}_{ij}^{0}(r)|,$ as averaged over all lines at a distance *r* from the disturbance and divided by its maximum value,

6 $${\rm{\Delta }}f({r}^{\ast },{t}^{\ast })=\langle {\rm{\Delta }}{F}_{kl}({r}^{\ast },{t}^{\ast })\rangle /\mathop{{\rm{\max }}}\limits_{t}(\langle {\rm{\Delta }}{F}_{kl}({r}^{\ast },{t}^{\ast })\rangle \mathrm{).}$$

**Figure 4:**
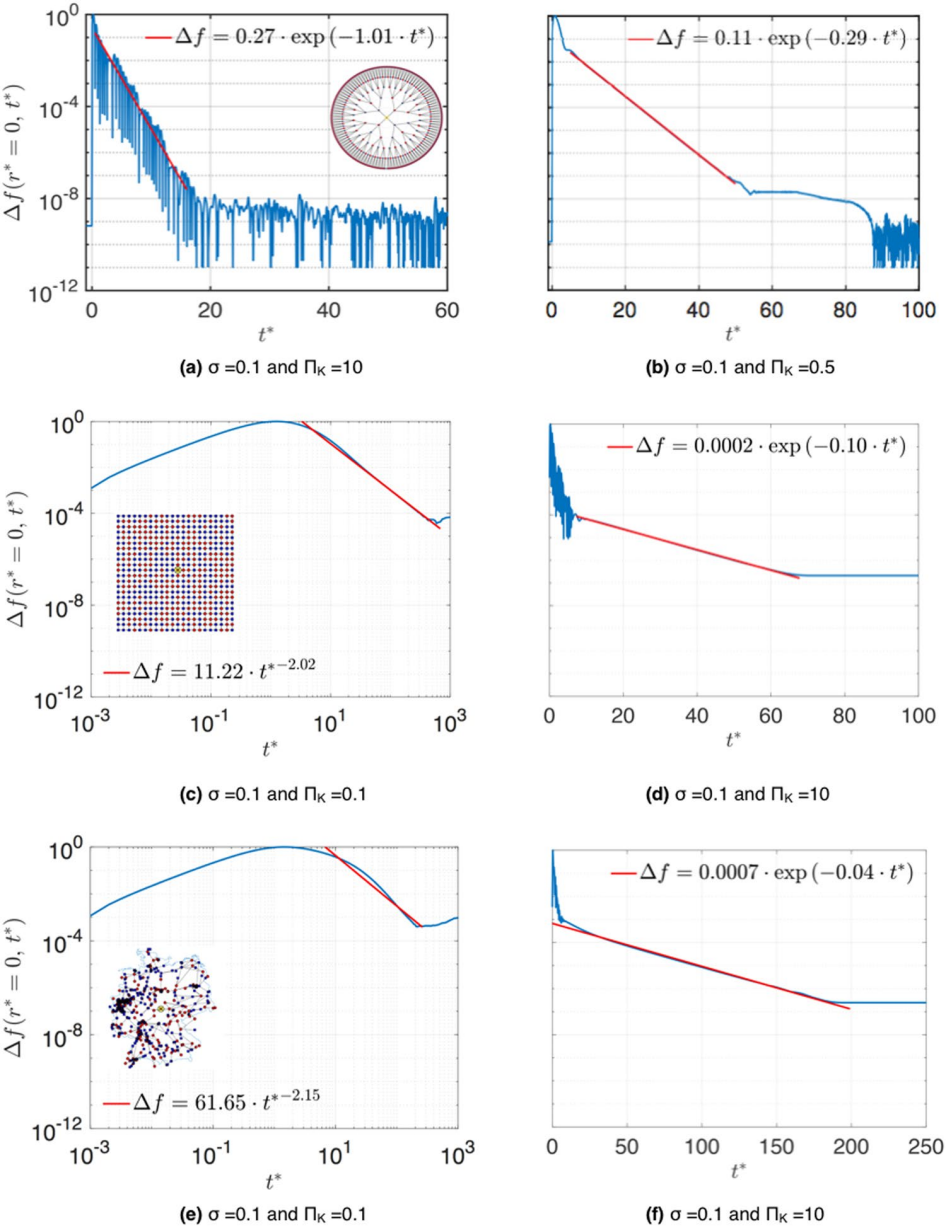
Power flow change Δ*f* (blue) as function of time t^*^ = *t*/*τ* at r^*^ = 0, disturbance *δP* = 0.001*K* for (**a**),(**b**) Cayley tree, (**c**), (**d**) square grid and (**e**), (**f**) German transmission grid with random arrangement of generators and motors. The transients are fitted to exponential and power law functions (red) as indicated.

**Figure 5:**
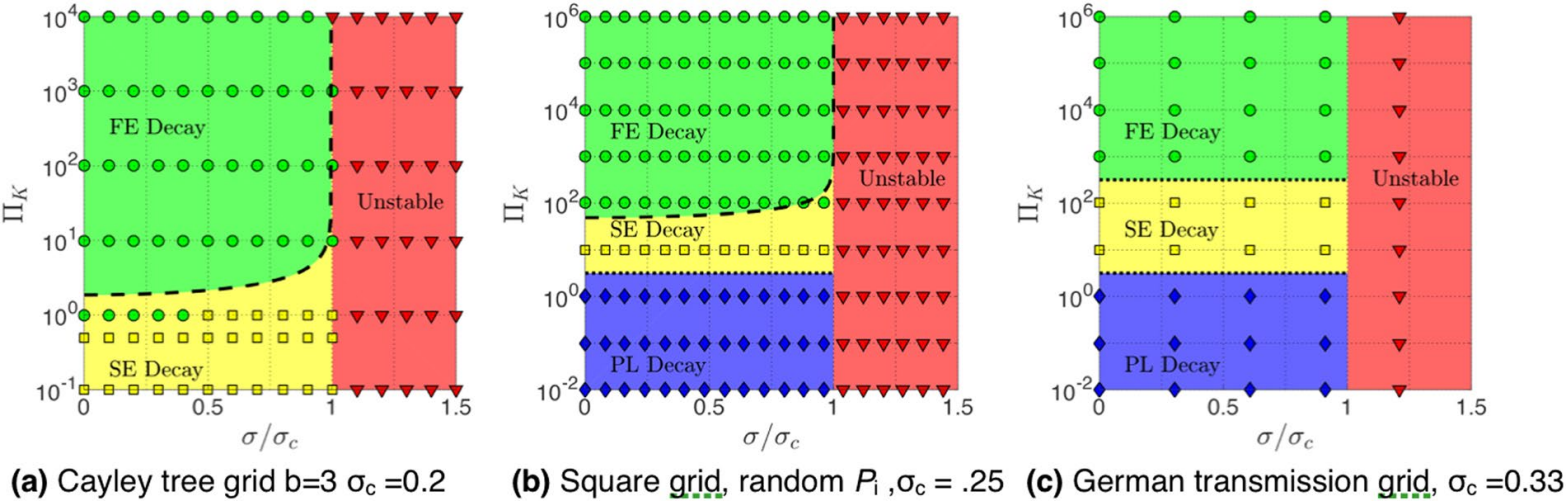
Phase diagrams as function of Π_*K*_ and *σ*/*σ*_*c*_. Numerically verified parameters that make the grid unstable (Red triangles), with fast exponential (FE) (Green circles), with slow exponential (SE) (Yellow squares) and power law (PL) decay (Blue diamonds). Red, green and yellow shaded regions are unstable (red), have FE (green), SE (yellow), PL (blue) decay. Dashed black line in figure (**a**) phase boundary between FE and SE decay, equation (16). Dashed black line in figure (**b**) equation (11). Dotted black lines in figures (**b**) and (**c**): numerical results.

**Figure 6:**
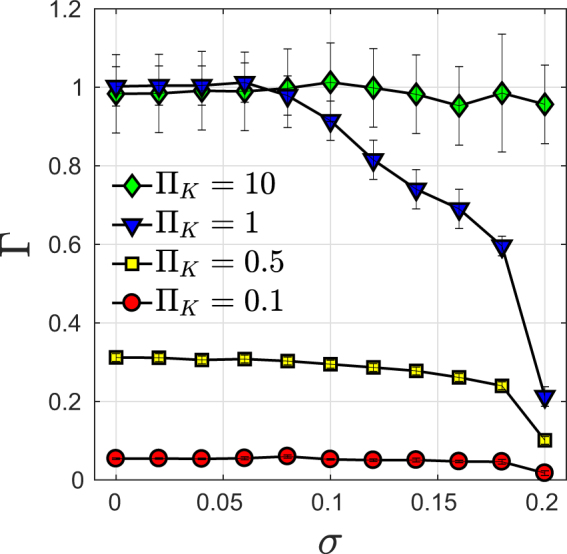
Decay rate Γ(*σ*) in units of Γ_0_ in the *b* = 3 Cayley tree, obtained by fitting transients with exponential decay.

In Fig. 4 we show examples for transient dynamics Δ*f* (r^*^, t^*^) in three grid topologies for different values Π_*K*_, *σ*. We next varied parameters Π_*K*_ and *σ* in small steps. Identifying all parameters with unstable solutions, we find unstable parameter regions $\sigma  > {\sigma }_{c},$ shown in red for the tree grid in Fig. 5(a), in (b) for the square grid with random arrangement of consumers and generators (the result for a periodic arrangement is shown in Supplementary Fig. 3), and in Fig. 5(c) for the German transmission grid. The critical values of *σ*_*c*_ are found to depend on grid topology: *σ*_*c*_ = 0.2 for the Cayley tree grid, *σ*_*c*_ = 2.00 for the square grid with periodic arrangement, *σ*_*c*_ = 1.25 with random arrangement and *σ*_*c*_ = 0.33 for the German transmission grid. In stable regions we identify three qualitatively different transient behaviors: The perturbation may decay *exponentially fast* (FE) with local relaxation rate Γ_0_ superimposed by oscillations, as seen in Fig. 4(a) in the tree grid. All parameter sets showing FE behavior are plotted in Fig. 5(a–c) as green circles. More examples for fast exponential transients are shown in Supplementary Fig. 2(d–f). Secondly, we observe exponential decay with a smaller relaxation rate Γ < Γ_0_ for a large interval of time $t-{t}_{0}\gg \tau$ as seen in Fig. 4(b) for the tree grid, d) for the square grid and f) for the German grid for the parameter sets shown in the phase diagrams Fig. 5(a–c) as yellow squares. Green shading denotes areas where fast relaxation (FE) is expected to occur, as limited by the dashed lines in Fig. 5(a,b), corresponding to the analytical result, equation (16) for the tree grid and to equation (11) for the square grids, respectively. For the German grid, we indicate the boundary of that region in Fig. 5(c) by a dotted line, according to numerical results. The yellow shaded areas in Fig. 5(a,b) show where slow relaxation (SE) is expected to occur, in good agreement with the analytical line. The slight inconsistency is in a region where we observe only small deviations of Γ from Γ_0_ well within the estimated error bars of the numerical results. We plot the parametric dependence of the relaxation rate Γ(*σ*) in Fig. 6 for *b* = 3 Cayley tree in units of Γ_0_, as obtained by fitting the transients with an exponential decay (error bars denote the range of Γ which provided good fitting to an exponential decay). Thirdly, we observe an even slower decay with a *power law* in time in square grids and in the German grid, as seen in Fig. 4(c) for a square grid and Fig. 4(e) for the German grid. That power is found to be close to 2, which is in very good agreement with the diffusive behavior of the change in power flow Δ*f*(*t*) obtained for a square grid by an analytical derivation^18^, as reviewed in the next section, equation (13). The numerical results indicate that such diffusive behavior may occur also in other meshed grids, as the example of the German grid at small values of the parameter Π_*K*_ < 2 shows, Fig. 4(e). Thus, as the inertia *J* and thereby Π_*K*_ is lowered, more nodes become correlated and the spreading of a disturbance is slowed down to a collective diffusive spreading for times $t > \tau$.

### Spatial Propagation of the Disturbance

**Figure 7:**
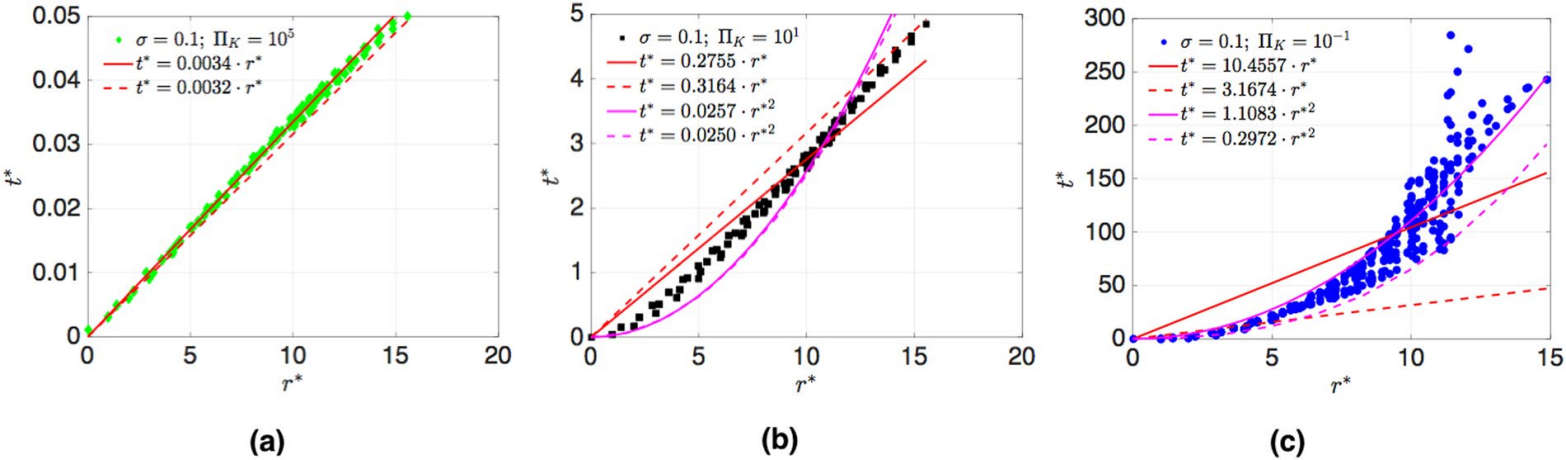
Time t^*^ = *t*/*τ* after initial disturbance *δ*Π = 0.001Π_*K*_, occurring at *t*_0_ = 0*s*, which perturbation needs to reach nodes at geometrical distance r^*^ = *r*/*a* in square grid (*L* = 22*a*) with random arrangement of generators and motors, *σ* = 0.1, (**a**) for Π_*K*_ = 10^5^, fitted to ballistic equation t^*^ = r^*^/(*vτ*/*a*) (red line), and analytical with equation (12) (dashed red line). (**b**) for Π_*K*_ = 10. (**c**) for low inertia Π_*K*_ = 0.1 fitted with ballistic (red line) and with diffusive formula t^*^ = r^*2^/(4*D*) (pink line). We show analytical curves for ballistic (red dashed line) and diffusive spreading (pink dashed line).

Next, we examine the spatial propagation of disturbances. If it propagates ballistically, it moves with velocity *v*, so that it reaches a node at distance *r* after time *t* according to *r* = *v*(*t* − *t*_0_), where *t*_0_ is the time when it occurs first. Diffusion corresponds to areal spreading, so that a node at distance *r* is reached according to *r*^2^ = 4*D*(*t* − *t*_0_) with diffusion constant *D*. Localization occurs when the disturbance does not spread beyond a certain localization length *ξ*, never reaching nodes at distances $r > \xi \mathrm{.}$ Let us start by analyzing the propagation in square grids with *L* = 22 and random arrangement of generators and motors. In Fig. 7 we show numerical results for *σ* = 0.1 for the arrival time t^*^ = *t*/*τ* (after initial disturbance *δ*Π_*P*_ = 0.001Π_*K*_ occurs at time *t*_0_ = 0*s*), which the power disturbance needs to reach nodes at geometrical distance r^*^ = *r*/*a*, where $r={({r}_{x}^{2}+{r}_{y}^{2})}^{\mathrm{1/2}}$ (results for distances *r*, defined as the minimal number of links connecting two nodes, are shown in the Supplementary). We define the arrival time as the time, when the phase deviation *α*_*i*_ exceeds a threshold *α*_*th*_ = 10^−6^*δ*Π_*P*_ at a node at distance *r*. In Fig. 7(a) we plot results for Π_*K*_ = 10^5^ together with t^*^ = cr^*^, where *c* = 0.0034 is fitted (red line). According to the analytical result it propagates for ${{\rm{\Pi }}}_{K} > {{\rm{\Pi }}}_{K}^{s}$ ballistically with the velocity given by equation (12), as derived in the next section. We plot t^*^ = r^*^/(*vτ*/*a*), as the dotted red line. Thus, we can confirm quantitative agreement with ballistic motion. Here, ${{\rm{\Pi }}}_{K}^{s}$ is given for the square grid by equation (11), as plotted in the phase diagram Fig. 5, the dashed line. b) for smaller inertia, Π_*K*_ = 10, we observe a crossover behavior where the data in Fig. 7(b) fits neither ballistic nor diffusive propagation well. For Π_*K*_ = 0.1, we find in Fig. 7(c), that the arrival time agrees with diffusive motion, t^*^ = cr^*2^ where we fitted *c* = 1.1083 (pink line), while the ballistic formula (straight lines) do not fit the data. Using the analytical result for the arrival time t^*^ = r^*2^/(4*D*)*f*_*th*_(*r*), where *f*_*th*_(*r*) is a logarithmic correction, which depends on threshold value *α*_*th*_, as derived in Supplementary III, we find that it provides a good lower bound to the data (pink dashed line).

**Figure 8:**
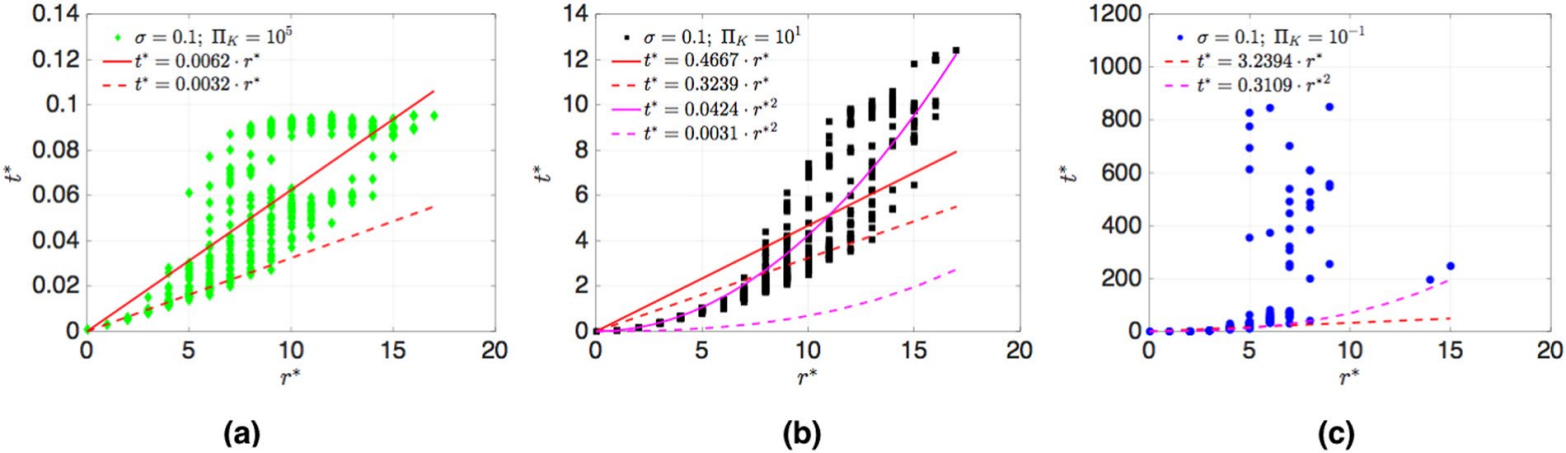
Time t^*^ = *t*/*τ* a disturbance needs to propagate distance r^*^ = *r*/*a* for german transmission grid (*σ* = 0.1), (**a**) for Π_*K*_ = 10^5^ fitted with t^*^ = cr^*^, *c* = 0.0062 (red line), and compared with *c* = 1//(*vτ*/*a*) with velocity *v*, equation (12) (dashed red line). For (**b**) Π_*K*_ = 10 we fit the data with the ballistic (red line) and with the diffusive formula t^*^ = cr^*2^, *c* = 0.0424 (pink line). For comparison, we show analytical results for ballistic (red dashed line) and diffusive spreading (pink dashed line). (**c**) for low inertia Π_*K*_ = 0.1 we show analytical curves for ballistic (red dashed line) and diffusive motion (pink dashed line).

In Fig. 8 we show for the german transmission grid the time t^*^ = *t*/*τ* which a power disturbance, initially at the node marked by a cross in Fig. 1, needs to reach a node at distance r^*^ = *r*/*a*. For *σ* = 0.1 and Π_*K*_ = 10^5^ we fit the numerical results with the ballistic formula t^*^ = cr^*^ with slope *c* = 0.0062 (red line), and compare it with the analytical value for velocity *v*, equation (12) (dashed red line). We see that the analytical formula provides a lower bound to the data, yielding evidence for ballistic motion for some nodes, until the node distance *r* = 9, which corresponds to the shortest path length from the source of the disturbance to the boundary of the german grid. The disturbance is seen to reach other nodes later, indicating anisotropy in the german grid. For (b) Π_*K*_ = 10 we show the fitted curves for ballistic (red line) and diffusive motion (pink line) and analytical curves (red and pink dashed lines). While diffusive spreading is fitting some nodes, the disturbance needs more time to reach other nodes, which is another consequence of anisotropy. For (c) Π_*K*_ = 0.1, corresponding to low inertia in the grid, we show the analytical curves for ballistic (red dashed line) and diffusive motion (pink dashed line). The scatter of the results for different nodes at distance *r* is too large for a meaningful fit. The analytical formula for diffusive spreading is seen to provide a good lower bound for the data. Thus, we find strong indications in the german grid that for low inertia the collective dynamics of nodes results in diffusive spreading of the disturbance. The spreading is more strongly delayed for some nodes, and there are indications of localization of disturbances in certain directions since some nodes do not become excited above the threshold within the observation time. We also note that for some nodes the relaxation to a stationary state did not take place before the signal started, see the Supplementary material for more details.

**Figure 9:**
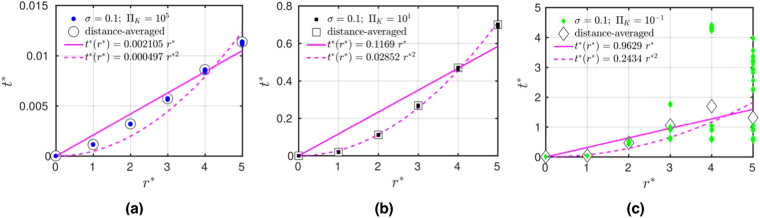
The time t^*^ = *t*/*τ* the power disturbance needs to reach a node at distance r^*^ = *r*/*a* for exemplary sets of parameters in a Cayley tree grid. The initial disturbance *δ*Π = 0.001Π_*K*_ occurs at *t*_0_ = 0*s*.

On the Cayley tree grid the time t^*^ = *t*/*τ* the power disturbance needs to reach a node at distance r^*^ = *r*/*a* for power ratio *σ* = 0.1 is shown in Fig. 9. For a) Π_*K*_ = 10^5^ we fitted the numerical results with the ballistic formula t^*^ = r^*^/(*vτ*/*a*) (pink line), and the quadratic formula (dashed pink line). As shown in the next section, the quadratic dependence t^*^ = cr^*2^, where *c* is a constant, is due to the quadratic dispersion of the Eigen modes of the generalized Laplacian on the tree grid. It fits for b) Π_*K*_ = 10 the numerical data even better. Note that this diffusion like dependence occurs at values of Π_*K*_, where we observed fast exponential decay. This is explained in the next section. Note also that for both Π_*K*_ = 10^5^ and Π_*K*_ = 10 the spreading is isotropic in all directions, as the comparison between node resolved (full symbols) and same distance *r* node averaged (white symbols) shows. As the inertia is lowered, there appears a strong anisotropy at large distance, however, as the results for Π_*K*_ = 0.1 show. Thus, we find for small inertia anisotropic spreading, while the disturbance still decays exponentially in time. This is explained in the next section by a topologically protected spectral gap in treelike grids.

### Linear Response theory and Spectral Analysis of Disturbances: Analytical results

For small perturbations, corresponding to the condition *σ* < σ^*^(*α*), we analyze the swing equation (4) by expanding it in the perturbation *α*_*i*_ − *α*_*j*_. This yields the linear wave equations on the grid^18^,

7 $${\tau }^{2}{\partial }_{t}^{2}{\alpha }_{i}+2\tau {\partial }_{t}{\alpha }_{i}+\sum _{j}{t}_{ij}({\alpha }_{i}-{\alpha }_{j})=\delta {{\rm{\Pi }}}_{i}(t),$$

with coupling amplitudes ${t}_{ij}={{\rm{\Pi }}}_{Kij}\,\cos ({\theta }_{i}^{0}-{\theta }_{j}^{0}),$ depending both on grid topology and on initial distribution of power *P*_*i*_ through stationary phases ${\theta }_{i}^{0}\mathrm{.}$ These coupling amplitudes define the generalized Laplace operator Λ with Λ_*ij*_ = −*t*_*ij*_ and Λ_*ii*_ = ∑_*i*_*t*_*ii*_, which is related to the stability matrix in linear stability analysis^21,25^ as used in small signal stability analysis^26,27^. We expand the phase deviation *α*_*i*_(*t*) and the disturbance *δ*Π_*i*_(*t*) = *JδP*_*i*_(*t*)/(*γ*^2^*ω*) in a generalized Fourier series in terms of the Eigenvectors *ϕ*_*n*_ of Laplacian matrix Λ, as defined by Λ*ϕ*_*n*_ = Λ_*n*_*ϕ*_*n*_, where Λ_*n*_ is its Eigenvalue^18,21,25,28^, see Supplementary III. For a local perturbation at a site *j*, lasting a short time interval ${\rm{\Delta }}t\ll \tau$ around time *t*_0_, *δ*Π_*i*_(*t*) = *δ*Π*δ*_*ij*_*τδ*(*t* − *t*_0_), we find

8 $${\alpha }_{i}(t > {t}_{0})=-\frac{\delta {\rm{\Pi }}}{2}\sum _{n}{{\varphi }}_{ni}{{\varphi }}_{ni}^{\ast }\frac{1}{\sqrt{1-{{\rm{\Lambda }}}_{n}}}({e}^{-{{\rm{\Omega }}}_{n+}(t-{t}_{0})}-{e}^{-{{\rm{\Omega }}}_{n-}(t-{t}_{0})}),$$

where ${{\rm{\Omega }}}_{n\pm }=-\,\mathrm{(1}\pm \sqrt{1-{{\rm{\Lambda }}}_{n}}\mathrm{)1/}\tau \mathrm{.}$ This formula relates the transient dynamics to the Eigenvalues ${{\rm{\Lambda }}}_{n}={\varepsilon }_{n}^{2}{\tau }^{2}$ and Eigenvectors *ϕ*_*n*_ of the Laplacian, which can be obtained numerically by exact diagonalization for arbitrary grids. For particular grids we obtained analytical solutions as summarized in the following.

#### Periodic Square Lattice

**Figure 10:**
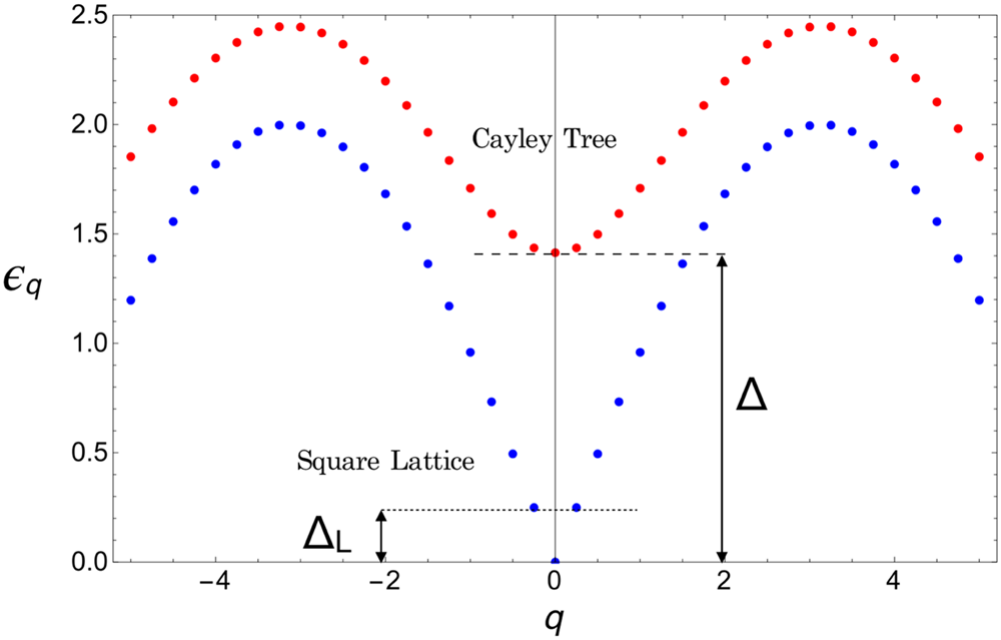
Dispersion of Eigenvalue *ε*_*q*_ of discrete wave equation (7) as function of wave number *q*_*n*_ for finite Cayley tree grid (red dots) and square lattice (blue dots). Δ is the spectral gap to the first excitation frequency in the Cayley tree grid (dashed line). For the square lattice the gap ${{\rm{\Delta }}}_{L}={\varepsilon }_{{q}_{1}}$ (dotted line) decreases with system size *L*.

For square lattices where *P*_*i*_ = ±*P* are arranged periodically, an analytical solution of the stationary power balance equation is obtained with plain wave Ansatz ${{\varphi }}_{qi}={c}_{q}{e}^{i{\bf{q}}{{\bf{r}}}_{i}}$ with wave vector **q**. The Eigenfrequencies ${\varepsilon }_{{q}_{n}}$ are^18^,

9 $${\varepsilon }_{{q}_{n}}=\sqrt{{{\rm{\Pi }}}_{K}}{\mathrm{(1}-{\sigma }^{2}/{\sigma }_{c}^{2})}^{\mathrm{1/4}}\sqrt{4-{f}_{{q}_{n}}}{{\rm{\Gamma }}}_{0},$$

where *σ*/*σ*_*c*_ = *P*/(4*K*) and ${f}_{{q}_{n}}=\mathrm{2(}\,\cos \,{q}_{nx}a+\,\cos \,{q}_{ny}a\mathrm{).}$ For finite size *L*, the wave vectors are quantized, *q*_*x*,*yn*_ = *n*_*x*,*y*_*π*/*L*, with *n*_*x*,*y*_ = 0, ±1, ... . The resulting dispersion of the Eigenfrequency *ε*_*q*_ is plotted in Fig. 10 as function of discrete wave numbers *q*_*n*_ (blue dots). We observe a spectral gap to the first excitation energy ${\varepsilon }_{{q}_{1}}={{\rm{\Delta }}}_{L}$ as indicated by the dotted line in Fig. 10,

10 $${{\rm{\Delta }}}_{L}={{\rm{\Pi }}}_{K}^{\mathrm{1/2}}{\mathrm{(1}-{\sigma }^{2}/{\sigma }_{c}^{2})}^{\mathrm{1/4}}\frac{\pi a}{L}{{\rm{\Gamma }}}_{0},$$

decreasing with size *L*. Applying linear response theory for small disturbances, inserting ${{\rm{\Lambda }}}_{n}={\tau }^{2}{\varepsilon }_{{q}_{n}}^{2}$ into the Fourier expansion, equation (8), we get the phase deviation *α*(*t*) for all times *t*. The condition that slow modes with small relaxation rate Γ < Γ_0_. Appear is, that the spectral gap Δ_*L*_ is smaller than the local relaxation rate, Γ_0_. This yields the parametric condition ${{\rm{\Pi }}}_{K} < {{\rm{\Pi }}}_{K}^{S}(L),$ where ${{\rm{\Pi }}}_{K}^{S}(L)$ depends on grid size *L* and power ratio *σ* as

11 $${{\rm{\Pi }}}_{K}^{S}(L)={\mathrm{(1}-{\sigma }^{2}/{\sigma }_{c}^{2})}^{-\mathrm{1/2}}{(\frac{L}{\pi a})}^{2}\mathrm{.}$$

This result is plotted in phase diagram Fig. 5(b) (dashed line) together with numerical results. If condition ${{\rm{\Pi }}}_{K} > {{\rm{\Pi }}}_{K}^{S}(L)$ is fulfilled, the disturbance moves ballistically with velocity

12 $$v=\sqrt{{{\rm{\Pi }}}_{K}{\mathrm{(1}-{\sigma }^{2}/{\sigma }_{c}^{2})}^{\mathrm{1/2}}}a/\tau \mathrm{.}$$

On the other hand, when the condition ${{\rm{\Pi }}}_{K} < {{\rm{\Pi }}}_{K}^{S}(L)$ is fulfilled, Eigen modes with small wave number *q* have purely imaginary complex Eigenfrequency Ω_*q*_ which results in slow decay of the phase deviations without any oscillations. Summing over all slow modes in the spectral representation of *α*_*i*_(*t*), equation (8) we find that a perturbation at node *j* propagates for times $t > \tau$ and distances exceeding the mean free path *l* = *vτ* diffusively with diffusion constant *D* = *v*^2^*τ*, see Supplementary III for the derivation. Diffusion causes slow power law relaxation of the disturbance at the initial site and an initial increase, followed by a power law decay at other sites. The resulting power law relaxation of the change in transmitted power between nodes *k* and *l* is^18^

13 $$\delta {F}_{kl}(t)=\pm \delta P{A}_{kl}\frac{{\pi }^{2}{a}^{2}}{{\omega }_{0}D{t}^{2}}\exp (-\frac{{({{\rm{r}}}_{{\rm{j}}}-{{\rm{r}}}_{{\rm{l}}})}^{2}}{4Dt})\mathrm{.}$$

Thus, we find that the change in transmitted electric power at the site of perturbation *r*_j_ decays with a power law in time with power 2 in excellent agreement with the numerical results for the periodic square grid, Fig. 4(c). The position of the maximum of the disturbance spreads according to ${r}_{max}^{2}=4Dt$ with time. Note that *r* is here defined as the geometrical distance $r={({r}_{x}^{2}+{r}_{y}^{2})}^{\mathrm{1/2}}$. After Thouless time *t*_*L*_ = *L*^2^/*D*^29^ the disturbance reaches the grid boundary and is reflected. Then, for times exceeding *t*_*L*_ the disturbance decays exponentially with rate ${{\rm{\Gamma }}}_{min}=\mathrm{(1}-{\mathrm{(1}-{\tau }^{2}{{\rm{\Delta }}}_{L}^{2})}^{\mathrm{1/2}}){{\rm{\Gamma }}}_{0}\mathrm{.}$ For square grids with inhomogenous distribution of power *P*_*i*_ slowly decaying modes appear when ${{\rm{\Pi }}}_{K} < {{\rm{\Pi }}}_{K}^{s}(L),$ with ${{\rm{\Pi }}}_{K}^{s}(L)$ given by equation (11), where *σ*_*c*_ is the critical value for that distribution of power *P*_*i*_. Diffusion occurs with an accordingly modified diffusion constant *D*. We plot ${{\rm{\Pi }}}_{K}^{s}(L),$ dashed line in Fig. 5(c), together with numerical results where we use the numerically obtained value for *σ*_*c*_.

#### Cayley Tree Grid

On a Cayley tree grid every inner node is connected to *d* = *b* + 1 other nodes, as shown in Fig. 1(a) for *b* = 3. For the Cayley tree with branching $b > 1$ symmetric eigenvectors were found in ref.^30^. With these Eigenvectors we obtain the Eigenfrequencies of the discrete wave equation 7 as

14 $${\varepsilon }_{q}=\sqrt{{{\rm{\Pi }}}_{K}}{(1-\frac{{\sigma }^{2}}{{\sigma }_{c}^{2}})}^{\mathrm{1/4}}\sqrt{b+1-2\sqrt{b}\,\cos \,qa}{{\rm{\Gamma }}}_{0}\mathrm{.}$$

For $b > \mathrm{1,}$ *q* can not be identified with a wave number since the phase of the Eigenvectors depends nonlinearly on *qa*. We plot *ε*_*q*_ in Fig. 10 for a finite sized tree as function of discrete number *q*_*n*_. It is remarkable that Eigenfrequencies equation (14) have for $b > 1$ a finite gap Δ, independent on the number of nodes *N*,

15 $${\rm{\Delta }}={{\rm{\Pi }}}_{K}^{\mathrm{1/2}}{\mathrm{(1}-{\sigma }^{2}/{\sigma }_{c}^{2})}^{\mathrm{1/4}}\sqrt{b+1-2\sqrt{b}}{{\rm{\Gamma }}}_{0},$$

indicated by the dashed line in Fig. 10. The condition that slow modes with Γ < Γ_0_, appear is Δ < Γ_0_, which yields the parametric condition ${{\rm{\Pi }}}_{K} < {{\rm{\Pi }}}_{K}^{s},$ with

16 $${{\rm{\Pi }}}_{K}^{s}={\mathrm{(1}-{\sigma }^{2}/{\sigma }_{c}^{2})}^{-\mathrm{1/2}}{(b+1-2\sqrt{b})}^{-1}\mathrm{.}$$

If that condition is fulfilled, the perturbation decays for large times exponentially with a reduced relaxation rate Γ_*min*_ = (1 − (1 − *τ*^2^Δ(*z*, Π_*K*_)^2^)^1/2^)Γ_0_ which can be much smaller than the local relaxation rate Γ_0_. The spatial spreading is diffusive, *r*^2^ = 4*D*_*b*_*t* with diffusion constant ${D}_{b}=\tau {{\rm{\Delta }}}^{2}\sqrt{b}/\sqrt{b+1-2\sqrt{b}}$. For ${{\rm{\Pi }}}_{K} > {{\rm{\Pi }}}_{K}^{s},$ the perturbation decays fast exponentially with local rate Γ_0_ = 1/*τ*. Due to the quadratic dispersion of *ε*_*q*_ for a Cayley tree, at small values of *q*, see Fig. (10), the spatial spreading scales with *r*^2^/(*ct*) where $c={\rm{\Delta }}\sqrt{b}/\sqrt{b+1-2\sqrt{b}}$. We plot ${{\rm{\Pi }}}_{K}^{s}(\sigma /{\sigma }_{c})$ in Fig. 5(a) as the dashed line. For a tree grid with inhomogenous distribution of power *P*_*i*_, typically, the slowly decaying modes are expected to appear when ${{\rm{\Pi }}}_{K} < {{\rm{\Pi }}}_{K}^{s},$ with ${{\rm{\Pi }}}_{K}^{s}$ given by equation (16), where *σ*_*c*_ is the critical value for that distribution of power *P*_*i*_.

## Discussion

In conclusion, we studied how the relaxation and propagation of disturbances in AC grids is modified when system parameters like the inertia in the grid are changed. To this end we solved the nonlinear dynamic power balance equations on three different grid topologies numerically and analyzed the results comparing them quantitatively with analytical insights obtained by linear response theory and spectral analysis. By a generalized Fourier expansion for the square grid and the Cayley tree grid we show that the long time transient behavior is governed by the spectral gap between the stationary state and the lowest Eigenmode of its grid. The Cayley tree grid has a finite spectral gap, which is protected by its topology and independent on grid size. Meshed grids, however are found to have a small spectral gap which is reduced strongly with increasing grid size, leading to slowed relaxation and collective diffusive propagation of disturbances. Analyzing the numerical results we confirm that, depending on inertia, geographical distribution of power, grid power capacity and topology, the disturbance may either decay exponentially in time with the decay rate of a single node, or exponentially with a smaller decay rate or, even more slowly, decaying with a power law in time, resulting in collective diffusive propagation. Let us discuss the relevance of our results for real grids like the german high voltage transmission grid, where the lines have a typical capacity of *K*_*ij*_ = 10 GW^19^. Assuming that half of the nodes act as generators and the other half as consumers to meet Germany’s peak power production of 83 GW^31^, we have |*P*_*i*_| = 300 MW. Typical conventional power plants of that rated power have inertia *J* = 10^4^ kg m^2^ and damping *γω*^2^ = 0.10*P*. This yields *Jω*^3^ = 310 GW, Π_P_ = 1.03 ⋅ 10^5^ and Π_K_ = 3.44 ⋅ 10^6^. Taking the condition ${{\rm{\Pi }}}_{K} < {{\rm{\Pi }}}_{K}^{S}(L),$ with equation (16) as an estimate that meshed grids show diffusive behaviour, that condition becomes for currently existing transmission grids, $L > 1856a\mathrm{.}$ Thus, diffusive propagation is unlikely to occur in present transmission grids even on the European scale. However, as conventional power plants become substituted by renewable energy resources the inertia in the grid is reduced substantially^1^. Thus, the dynamics of transmission grids will change. For very small inertia of *J* = 0.1 kg m^2^ and otherwise same parameters, we find Π_P_ = 1.03 and Π_K_ = 34.45 so that the condition to observe diffusion is $L > 5.87a$ and becomes relevant for transmission grids on the national scale. If no measures are undertaken to substitute the inertia of conventional power plants^32^, we conclude that the energy transition will change the transient dynamics drastically, disturbances will relax more slowly and spread by collective diffusive propagation, reducing the grid stability in meshed grids. Our results on tree grids suggest that a reduction of meshedness, may help to localize disturbances in the grid and yield faster relaxation, improving thereby the grid stability.

The linear response theory presented and applied here can be extended to larger perturbations by including nonlinear terms which introduce couplings between Eigenmodes of the Laplacian. This can be taken into account in various approximations, which will be considered in future research. Our results may also have important consequences for other systems modeled by a network of nonlinear oscillators, ranging from metabolic systems, to neuroscience and to supply chain networks. After submission of this manuscript, we learnt of a related work, which employs a linear response theory to calculate stability measures in 1st order Kuramoto models, corresponding to the swing model without inertia^33^.

## Electronic supplementary material

Propagation in a Cayley Tree Grid

Propagation in the German Transmission Grid

Propagation in a Square Grid

Scientific Reports Supplementary Propagation of Disturbances in AC Electricity Grids
