# Supplementary material for: Propagation of Disturbances in AC Electricity Grids
Source: Sci Rep. 2018 Apr 24;8:6459. doi: 10.1038/s41598-018-24685-5 (PMC5915393; doi:10.1038/s41598-018-24685-5)
Supplement: Supplementary file 4 — Scientific Reports Supplementary Propagation of Disturbances in AC Electricity Grids [file 41598_2018_24685_MOESM4_ESM.pdf]

Scientific Reports Supplementary
Propagation of Disturbances in AC Electricity Grids

Samyak Tamrakar[a,b], Michael Conrath[a], Stefan Kettemann[a,c]

[a] *Jacobs University, Department of Physics and Earth Sciences, Campus Ring 1, 28759 Bremen, Germany*
[b] *Institute of Physics, Carl von Ossietzky Universität Oldenburg, Ammerländer Heerstraße 114-118, 26129 Oldenburg*
[c] *Division of Advanced Materials Science, Pohang University of Science and Technology (POSTECH), San 31, Hyoja-dong, Nam-gu, Pohang 790-784, South Korea*

I. Numerical Simulations

We employ a standard differential equation solver, the Runge-Kutta algorithm [1] using the commercial software MATLAB®. Since this is a shooting method its convergence is improved considerably by setting the phases to the stationary state solutions before the perturbation. The perturbation is applied at $t = 0$. Calculated time spans prior and past perturbation were iteratively adjusted to ensure both a stationary state onto which the perturbation is applied and to capture the whole perturbation event until complete decay. Temporal resolution was chosen fine enough to avoid undersampling of the oscillating phases using the phase portraits $\dot{\alpha}_i(\alpha_i)$ as sensors. They would show smooth curves for sufficient resolution or angled curves for too poor resolution. In favour of automatized calculation, time span and resolution were mostly not adapted but rather chosen better than necessary. Concretely, for German and square grid, throughout all values of Π_K, $t \in [-1000\tau, 1000\tau]$ in steps of $10^{-3}\tau$. For the Cayley tree grid, time span and resolution were i) $t \in [-1000\tau, 1000\tau]$ in steps of $10^{-3}\tau$ for $\Pi_K < 10$ and ii) $t \in [-30\tau, 70\tau]$ in steps of $10^{-3}\tau$ for $\Pi_K \geq 10$.

In figure 1, we show phase portraits, plots of phase velocity $\dot{\alpha}_i$ versus phase α_i. We see in the upper figure that the disturbance remains within the basin of attraction of the attractive fixed point for $\Pi_K = 100$ and σ not exceeding 0.2. There is a slight shift of the phase to which the perturbation decays at the end, which we find to be due to a global phase shift of all nodes induced by the disturbance at the center node. In the lower figure it is seen that the perturbation destabilizes the grid when $\sigma = 0.22$, where the phase deviation increases unbounded. If there is no stable solution the phase perturbation increases without bound as seen in the example of Supp. figure 2 for the Cayley tree grid and in the lower phase portrait of Supp. figure 1.

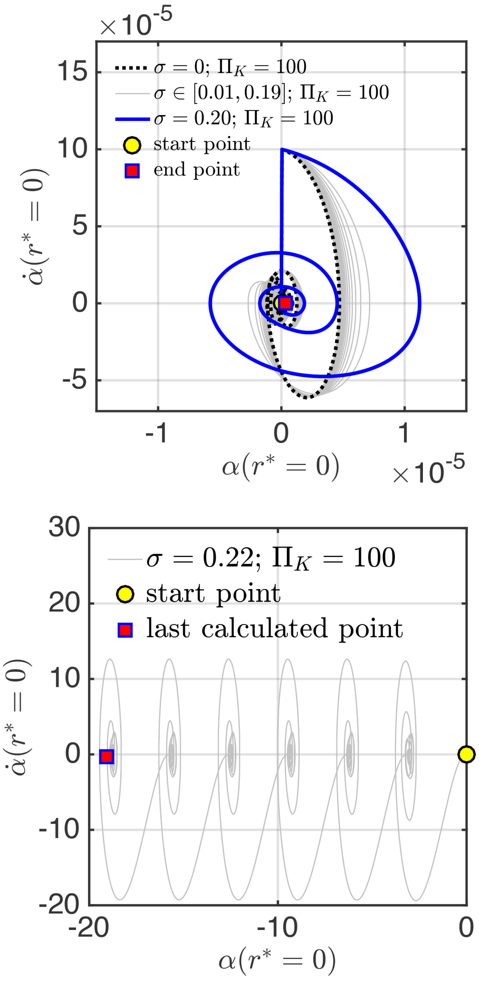

Figure 1: Phase portrait $\dot{\alpha}(r, t^*)$ versus $\alpha(r, t^*)$ for the $b = 3$ Caylee tree grid for $\Pi_K = 100$ and various σ.

II. Stability

Depending on the magnitude of the disturbance it can destabilize the grid already at smaller values of σ than the critical value σ_c above which there is no stationary solution, $\sigma < \sigma_c$. In order to get a typical upper limit for the size of the perturbation α before it kicks the system out of the stability, let us first disregard the dependence of the phase deviation at node i, α_i, on the perturbation at neighbored sites α_j. This reduces the swing equations equation M(4) of the main article to the one of a single damped, driven nonlinear pendulum. For large times $t \gg 0$ it is well known to have two stable solutions: *1. The stationary solution:* There is a stable fixed point at $\partial_t \alpha_i = 0$, $\alpha_i = n2\pi$, n integer, to which small deviations relax exponentially fast with the local decay rate $\Gamma_0 = 1/\tau$. *2. The over-swinging pendulum solution:* when the driving force and damping are in balance, the phase velocity oscillates around the value $\delta\omega_i = P_i/(2\gamma\omega)$.

There are saddle point solutions at $(\alpha_{si}, \partial_t \alpha = 0)$, where α_{si} is given by

$$\alpha_{si} = -2\mathrm{arctan}\left(\frac{\sum_{\mathrm{j}} \Pi_{\mathrm{Kij}} \cos(\theta_{\mathrm{i}}^0 - \theta_{\mathrm{j}}^0)}{\sum_{\mathrm{j}} \Pi_{\mathrm{Kij}} \sin(\theta_{\mathrm{i}}^0 - \theta_{\mathrm{j}}^0)}\right). \quad (1)$$

The condition for phase points to lie inside the stability region at node i is then obtained from this local stability analysis to be approximately given by [2]

$$\alpha_i^2 + \frac{(\tau \partial_t \alpha_i)^2}{(1 + \sqrt{1 - \sum_j \Pi_{Kij} \cos(\theta_i^0 - \theta_j^0 - \alpha_{si})})} \ll \alpha_{si}^2. \quad (2)$$

Thus, we can ensure stability against the perturbation $\alpha(t)$, by making sure that it satisfies the stability condition equation (2) for all times t. While that depends on the power distribution P_i and the topology of the grid through the stationary phase angles θ_i^0, we can get a typical upper limit for the allowed size of the perturbation α by substituting $\sin(\theta_i^0 - \theta_j^0)$ with the typical value of $P_i/(d_i K)$, which we denoted above by σ/σ_c. Substitution into equation (1) gives the saddle point value $\alpha_s = -2\mathrm{arcsin}(\sqrt{1 - \sigma^2/\sigma_c^2})$. Thus, for fixed perturbation amplitude α we find a critical value of σ, above which the disturbance causes instability,

$$\sigma^*(\alpha) = \sigma_c \cos(\alpha/2). \quad (3)$$

We see that the disturbance can destabilize the grid at smaller values $\sigma^*(\alpha) < \sigma_c$. $\sigma^*(\alpha)$ coincides with σ_c only in the limit, when the perturbation amplitude is vanishing, $\sigma^*(\alpha \to 0) = \sigma_c$.

III. Response to Disturbances: Analytical Theory

Stating from the linearized wave equation in the presence of a disturbance, obatined by a linear expansion in phase perturbation α_i in the presence of a fluctuation in power δP,

$$\tau^2 \partial_t \alpha_i + 2\tau \partial_t \alpha_i = \sum_j t_{ij}(\alpha_i - \alpha_j) + \frac{\delta P_i(t)}{J\omega}\tau^2 \quad (4)$$

we define the weighted Laplacian Λ with

$$\Lambda_{ij} = -t_{ij} \text{ and } \Lambda_{ii} = \sum_j t_{ij} \quad (5)$$

to obtain

$$\tau^2 \partial_t \vec{\alpha} + 2\tau \partial_t \vec{\alpha} + \Lambda \vec{\alpha} = \frac{\delta P_i(t)}{J\omega} \frac{J^2}{\gamma^2} \;. \quad (6)$$

Since $\Pi_P = (JP)/(\gamma^2 \omega)$, we can introduce

$$\partial \Pi_i = \frac{J \partial P_i(t)}{\gamma^2 \omega} \;. \quad (7)$$

Thus, we can write the phase deviation $\alpha_i(t)$ as a generalized Fourier series by writing its time dependence as a Fourier integral, and expanding its spatial dependence in terms of the Eigenvectors ϕ_n of the generalized Laplace operator Λ, defined by $\Lambda \phi_n = \Lambda_n \phi_n$, where Λ_n are its Eigenvalues[3, 2, 4]. Thereby we obtain[2, 5]

$$\alpha_i(t) = \int_{-\infty}^{\infty} d\epsilon \sum_n c_{n\epsilon} \phi_{ni} e^{-i\epsilon t}. \quad (8)$$

Expanding the disturbance likewise in a generalized Fourier series we get

$$\delta \Pi_i(t) = \int_{-\infty}^{\infty} d\epsilon \sum_n \eta_{n\epsilon} \phi_{ni} e^{-i\epsilon t}. \quad (9)$$

Now, we can insert both expansions into equation (9)M and find, requiring that the equation is fullfilled for each term of the Fourier series,

$$\left(-\tau^2 \epsilon^2 - \imath 2\tau\epsilon + \Lambda_n\right) c_{n\epsilon} = \eta_{n\epsilon}. \quad (10)$$

For given disturbance, the Fourier component of the phase deviation $c_{n\epsilon}$ is thus given in response to the one of the disturbance $\eta_{n\epsilon}$. Inserting that expression for $c_{n\epsilon}$ back into the Fourier series we get

$$\alpha_i(t) = \int_{-\infty}^{\infty} d\epsilon \sum_n \left(-\tau^2 \epsilon^2 - \imath 2\tau\epsilon + \Lambda_n\right)^{-1} \eta_{n\epsilon} \phi_{ni} e^{-i\epsilon t}. \quad (11)$$

The integral over the angular frequency ϵ can be performed by means of the residuum theorem, noting that there are two poles in the lower complex plane, $\epsilon_{n\pm} = -\imath(1 \pm \sqrt{1 - \Lambda_n})1/\tau$. We note that the coefficients $\eta_{n\epsilon}$ are complex and depend on ϵ. If the disturbance sets in at time t_0, $\eta_{n\epsilon}$ has a phase factor $\exp(\imath\epsilon t_0)$. Thus, for $t > t_0$, the integrand is convergent in the lower complex plane, so that we can close the integration contour there, and obtain

$$\begin{aligned} \alpha_i(t > t_0) = -\frac{\pi}{\tau} \sum_n \phi_{ni} \frac{1}{\sqrt{1 - \Lambda_n}} \\ \times \left(\eta_{n\epsilon_{n+}} e^{-i\epsilon_{n+}t} - \eta_{n\epsilon_{n-}} e^{-i\epsilon_{n-}t}\right). \end{aligned} \quad (12)$$

For $t < t_0$ the integrand is convergent in the upper complex plane, where there are no poles, so that the residuum is vanishing and we find $\alpha_i(t < t_0) = 0$. equation (12) is valid in linear order for any perturbation $\delta \Pi_i(t)$ and any electricity grid, inserting the Eigenvalues Λ_n and Eigenvector components ϕ_{ni} of the respective Laplacian.

For a local perturbation at a site j lasting only a short time interval $\Delta t \ll \tau$ around time t_0, we can choose the perturbation as $\delta \Pi_i(t) = \delta \Pi \delta_{ij} \tau \delta(t - t_0)$. Fourier transformation gives $\eta_{n\epsilon} = \frac{1}{2\pi} \delta \Pi \tau \phi_{nj}^* e^{\imath\epsilon t_0}$. Insertion into equation (12) gives then

$$\begin{aligned} \alpha_i(t > t_0) = -\frac{\delta \Pi}{2} \sum_n \phi_{ni} \phi_{nj}^* \frac{1}{\sqrt{1 - \Lambda_n}} \\ \times \left(e^{-i\epsilon_{n+}(t - t_0)} - e^{-i\epsilon_{n-}(t - t_0)}\right). \end{aligned} \quad (13)$$

Thus, it remains to find the Eigenvalues Λ_n and Eigenvector components ϕ_{ni}.

Square Grid. For a square grid with transmission line length a and power capacitance K and periodically arranged generator and consumer power $P_i = \pm P$, the Eigenvectors are plain waves with $\phi_{q_n i} = c_{q_n} e^{\imath \mathbf{q_n r}_i}$, where $\mathbf{q_n}$ is the wave vector which takes on a grid of finite size L discrete values, $\mathbf{q_n} = (n_x, n_y)\pi/L$, where $n_x, n_y \in \{-L/(2a), ..., +L/(2a)\}$. The Eigenvalues of the Laplacian Λ are given by $\Lambda_n = \tau^2 \epsilon_{q_n}^2$, where the Eigenfrequency ϵ_q of the linear wave equation is[2],

$$\epsilon_q = \sqrt{\Pi_K}(1 - \sigma^2/\sigma_c^2)^{1/4}\sqrt{4 - f_q}\Gamma_0, \quad (14)$$

where $\sigma/\sigma_c = P/(4K)$ and $f_q = 2(\cos q_x a + \cos q_y a)$. Insertion in equation (13) thus yields the phase pertubation in response to a change of the power at time t_0 at site j, $\delta\Pi_i(t)$ as

$$\alpha_i(t > t_0) = \delta\Pi \frac{1}{N} e^{-(t-t_0)/\tau} \sum_{n_x, n_y} e^{\imath \mathbf{q_n}(\mathbf{r_i} - \mathbf{r_j})} \times \frac{\sinh\left(\frac{t-t_0}{\tau}\sqrt{1-\Lambda_n}\right)}{\sqrt{1-\Lambda_n}}. \quad (15)$$

Thus, inserting all Eigenvalues $\Lambda_n = \tau^2 \epsilon_{q_n}^2$ with equation (14) we get the transient behavior of the phase deviation for all times $t > t_0$. For large momenta q, the ballistic limit, the relaxation is fast with the local rate Γ_0 and there is a real frequency with linear dispersion, $\Omega_q|_{q \ll 0} \approx -\imath\Gamma_0 + v_0 q$ with velocity $v_0 = \sqrt{\Pi_K}(1 - \sigma^2/\sigma_c^2)^{1/4} a/\tau$. At large time $t \gg \tau$ all such modes with Eigenvalues $\Lambda_n > 1$ have decayed.

Depending on the system parameters there can exist slow modes with $\Lambda_n < 1$, which decay with smaller rate $\Gamma_{n-} = \Gamma_0(1 - (1 - \Lambda_n)^{1/2})$. If this condition is fulfilled, Eigenmodes with small wave number q appear whose Eigenfrequency is purely imaginary, $\Omega_q = -\imath\Pi_K \cos(1 - \sigma^2/\sigma_c^2)^{1/2} a^2 \mathbf{q}^2$, which decay slowly without oscillations. Then, summing over all modes in the spectral representation of $\alpha_i(t)$, we find that an initially localized perturbation at node l spreads for times $t > \tau$ and distances exceeding the mean free path $l = v_0\tau$, $|r_\mathrm{i} - r_\mathrm{l}| > l$, using the continuum limit

$$\sum_{\vec{q}} \rightarrow \frac{1}{\left(\frac{2\pi}{L}\right)^2} \int \mathrm{d}\vec{q} \ , \quad (16)$$

according to

$$\alpha_i(t) = \delta\Pi \frac{1}{N} \frac{L^2}{4\pi^2} \int_{-\pi/a}^{+\pi/a} \mathrm{d}k_x \int_{-\pi/a}^{+\pi/a} \mathrm{d}k_y \times e^{\imath(k_x \hat{e}_{ijx} a + k_y \hat{e}_{ijy} a)} e^{-(t-t_0)D(k_x^2 + k_y^2)}, \quad (17)$$

yielding

$$\alpha_i(t) = \frac{\alpha_0 a^2}{4\pi D_0 t} \exp\left(-\frac{(\mathbf{r}_\mathrm{i} - \mathbf{r}_\mathrm{l})^2}{4D_0 t}\right). \quad (18)$$

Thus, the initially localized perturbation spreads diffusively with diffusion constant

$$D = v_0^2\tau = \Pi_K(1 - \sigma^2/\sigma_c^2)^{1/2} a^2/(\tau). \quad (19)$$

Diffusion causes very slow power law relaxation of the disturbance at the initial site, and an initial increase, followed by a slow power law decay at other sites. If we define a threshold value $\alpha_{th} = 10^{-p}\delta\Pi$, the time t the disturbance arrives at distance r is given by

$$t = \frac{r^2}{4D} \frac{1}{p - \ln(4\pi D t/(a^2))} \approx \frac{r^2}{4D} f_{th}(r), \quad (20)$$

where we iterated the equation once, to get the approximative expression in the last line with

$$f_{th}(r) = \frac{1}{p - \ln(\pi r^2/(pa^2))}. \quad (21)$$

Note that r is here the geometrical distance $r = (r_x^2 + r_y^2)^{1/2}$. The resulting power law relaxation of the change in transmitted power between nodes k and l is then obtained to be [2]

$$\delta F_{kl}(t) = \pm\delta P A_{kl} \frac{\pi^2 a^2}{\omega_0 D t^2} \exp\left(-\frac{(\mathbf{r}_\mathrm{i} - \mathbf{r}_\mathrm{l})^2}{4Dt}\right). \quad (22)$$

IV. Further Numerical Results

Transient Dynamics

In figure 2 we show further examples for transient dynamics, in particular, the results for square grids with periodic arrangement of consumers and generators are seen to be very similar to the ones with random arrangement shown in the main article, so that we can conclude that deep in the stable region $\sigma \ll \sigma_c$, the spatial distribution of power has a small influence on the transient dynamics. This can also be seen by looking at the phase diagram for square grids with periodic arrangement, which we show in figure 3.

Propagation of Disturbances

As mentioned in the main article, for low inertia, $\Pi_K = 0.1$, not all nodes in the german transmission grid equilibrated within the time interval of 1000τ, we had taken before the signal has been turned on. Those nodes are shown in figure 4.

The spatial propagation can also be measured by calculating the expectation value of the squared distance, as given by $\langle r_t^2 \rangle = \sum_i \alpha_i^2(t)(r_i - r_j)^2 / \sum_i \alpha_i^2(t)$. Diffusion results in linear increase with time for times $t - t_0 > \tau$, while ballistic motion gives a faster, quadratic increase. In figure 5 b) the result is shown for a square grid with periodic arrangement of P_i. We find that $\langle r_t^2 \rangle$ increases initially very fast for times $t - t_0 < \tau$.

For small $\Pi_K = 0.1$, corresponding to low inertia, we see the slowing down to a power law increase for $t - t_0 > \tau$ with $\langle r_t^2 \rangle \sim t^\beta$, where
$\beta = 1.12$ fits the data, which is another indication of diffusive propagation in square grids with small inertia. Finally, the expectation value of the squared distance converges to a value of the order of the system area L^2 for large times $t - t_0 > 1000\tau$. Arranging P_i randomly, we find an even better agreement with diffusive behavior, $\beta = 1.01$ for $\Pi_K = 0.1$. On the Cayley tree grid such a slow increase of $\langle r_t^2 \rangle$ is absent, it reaches a value of the order of the system area much earlier. This is in agreement with our observation that the disturbance decays exponentially fast in tree grids, figure 3 a).

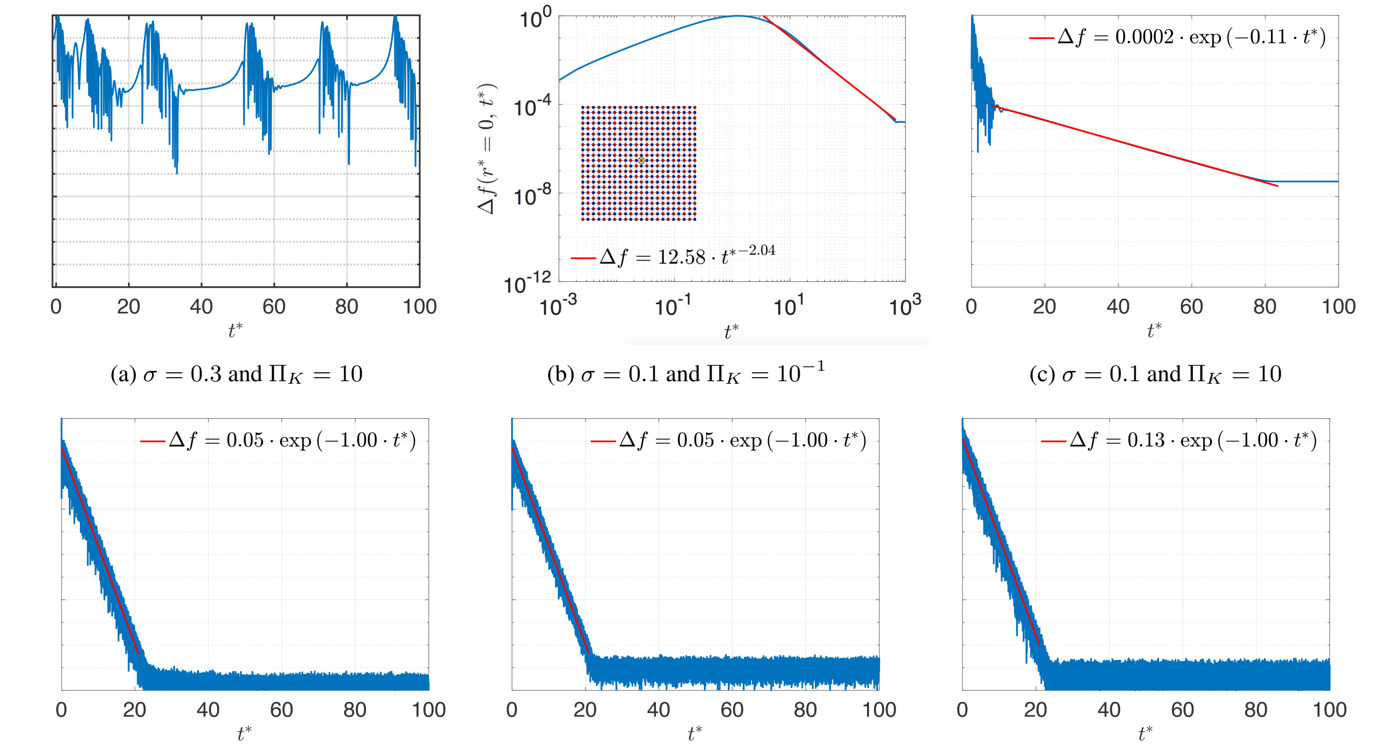

(a) $\sigma = 0.3$ and $\Pi_K = 10$ (b) $\sigma = 0.1$ and $\Pi_K = 10^{-1}$ (c) $\sigma = 0.1$ and $\Pi_K = 10$

(d) $\sigma = 0.1$ and $\Pi_K = 10^5$ (e) $\sigma = 0.1$ and $\Pi_K = 10^5$ (f) $\sigma = 0.1$ and $\Pi_K = 10^5$

Figure 2: Averaged change of power flow as function of time (Blue Curves) at $r^* = 0$ with disturbance in power $\delta P = 0.001P$ for (a) a Cayley tree grid $(N = 484)$ for a set of parameters where it is unstable, (b)-(d) a square grid, $L = 22$, with periodic arrangement, (e) a square grid, $L = 22$, with random arrangement, (f) German transmission grid with random arrangement of generators and motors. We fitted the numerical results (blue) with exponential and power law functions (red).

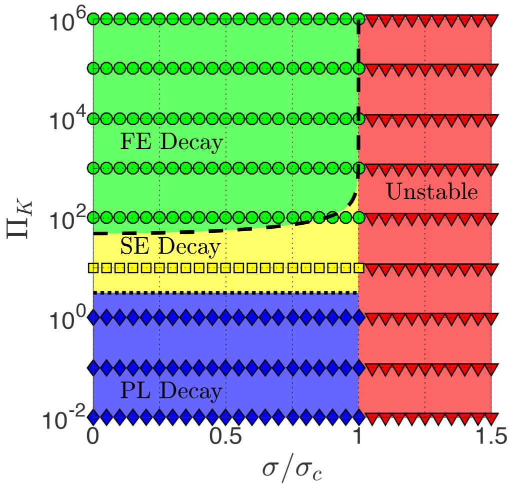

Figure 3: Phase diagram as function of parameters Π_K and σ for a square grid with periodic P_i, $L = 22$, $\sigma_c = 2.00$. Red triangle, green circle, yellow square and blue diamond represent numerically verified parameters that makke the grid unstable, that result in fast exponential (FE) decay, in slow exponential (SE) decay and in power law (PL) decay, respectively. Red, green and yellow shaded regions represent parameters that, according to analytical results, are unstable, to have FE decay and SE decay, respectively. Blue shading represents the numerically obtained region with PL decay. Dashed black line is the analytical result for the boundary, equation (8) of the main article. The dotted black line is a numerically obtained boundary.

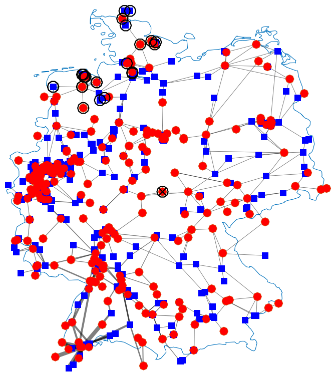

Figure 4: Nodes which did not equilibrate in the german grid at low inertia, corresponding to $\Pi_K = 0.1$ are indicated by circles.

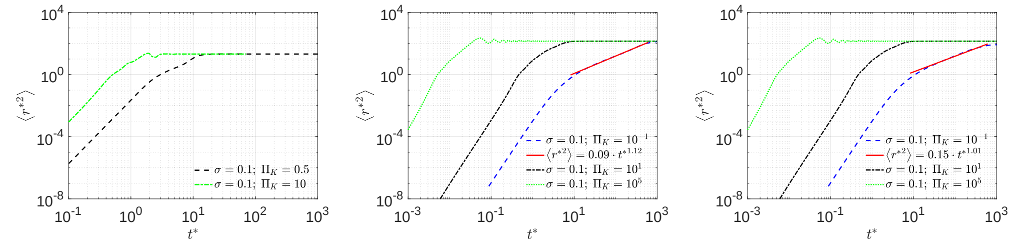

(a) (b) (c)

Figure 5: Expectation value of the square of the distance r_i to the origin of power disturbance $\delta P = 0.001K$ as function of time, for the exemplary sets of parameters given in the insets (a) in a Cayley tree grid ($N = 485$), (b) in a square grid ($L = 22$) with periodic arrangement, (c) in a square grid ($L = 22$) with random arrangement.

[1] Kreyszig, E. Advanced Engineering Mathematics, 9th edition., Wiley, New York (2006).

[2] Kettemann, S. Delocalization of disturbances and the stability of AC electricity grids. *Phys. Rev. E* 94, 062311 (2016).

[3] Nishikawa, T., Motter, A. Comparative analysis of existing models for power grid synchronization, New J. Phys. **17**, 015012 (2015).

[4] Coletta, T., Jacquod, P. Linear Stability and the Braess Paradox in Coupled-Oscillator Networks and Electric Power Grids, Phys Rev E **93**, 032222 (2016).

[5] Torres-Sánchez, L. A., Freitas de Abreu, G. T., Kettemann, S. Analysis of the Dynamics and Topology Dependencies of Small Perturbations in Electric Transmission Grids, subm. to IEEE Power Systems, arXiv:1706.10130 (2017).
